# A Retrospective Study on the Status of Working Equids Admitted to an Equine Clinic in Cairo: Disease Prevalence and Associations between Physical Parameters and Outcome

**DOI:** 10.3390/ani14050817

**Published:** 2024-03-06

**Authors:** Beatrice Benedetti, Francesca Freccero, Jill Barton, Farah Elmallah, Sandy Refat, Barbara Padalino

**Affiliations:** 1 Department of Agricultural and Food Sciences, University of Bologna, Viale Fanin 44, 40127 Bologna, Italy; beatrice.benedetti7@unibo.it; 2 Department of Veterinary Medical Sciences, University of Bologna, Via Tolara di Sopra 50, 40064 Bologna, Italy; francesca.freccero2@unibo.it; 3 Egypt Equine Aid, Badrshein, Giza 12989, Egyptfarahelmallah@hotmail.com (F.E.);; 4 School of Environmental and Rural Science, University of New England, Armidale, NSW 2351, Australia

**Keywords:** horse, donkey, euthanasia, Cairo, welfare, quality of life, suffering, end-of-life

## Abstract

**Simple Summary:**

Equids often live and work below welfare standards till exhaustion and die painfully. Euthanasia for humane reasons should be considered in some situations. Scientific evidence on the most common diseases of working equids is still limited. This study aimed to describe the population and disorders of the equids that arrived at the Egypt Equine Aid clinic (Cairo, Egypt) from 2019 to 2022. Secondly, it aimed to find any possible association between physical parameters at admission and outcome (i.e., discharged, euthanasia, or dead). The results of this study showed that working equids suffered mainly from wounds, orthopaedic problems, colics, and infectious diseases. In addition, in the univariable ordinal regression, some easy-to-assess physical parameters, namely the abnormal colour of the mucous membrane, highly increased capillary refill time, and increased heart rate, were associated with the non-survival outcome (i.e., euthanasia or dead). In conclusion, owner education and timely medical assistance in case of need remain the central focus to ensure a life worth living for working equids. This study to our best knowledge represents the first one to describe the disease prevalence of the working equid population around the Cairo area and to identify critical points to improve their quality of life.

**Abstract:**

Working equids are often used to exhaustion, living and dying in conditions below minimal welfare standards. Due to their poor welfare status, euthanasia should be considered in certain conditions. The study aimed to describe the population and the disease frequency of the working equids admitted at an equine clinic in Cairo (i.e., Egypt Equine Aid (EEA)) from 2019 to 2022 and identify possible associations between physical parameters at admission and the outcome. Records of 1360 equids admitted at EEA were reviewed. The majority of the admitted equids were horses (65.6%), followed by donkeys (33%), in particular stallions (68.7%), from 1 to 15 years old (74.8%). Hospitalisation was mainly due to wounds (28.9%), orthopaedic problems (27.4%), colic (8.5%), or infectious diseases (7.4%). The majority of the equids were discharged, but 5.1% died on their own, without human intervention, and 23% were euthanised. Text mining revealed the anamnesis’s most frequent words were ‘accident’, ‘lameness’, and ‘wound’. In addition, owners sometimes reported using inappropriate remedies (e.g., firing) before hospitalisation. Multivariable ordinal regression analysis performed between physical parameters and the outcome (ordered based on severity: discharged, euthanasia, and dead) revealed that sex (male vs. female: OR = 1.33; *p* < 0.05), colour of the mucous membrane (pathological vs. physiological: OR = 1.72; *p* < 0.01), and capillary refill time (pathological vs. physiological: OR = 1.42; *p* = 0.02) increased the likelihood of a non-survival outcome. In conclusion, early euthanasia should be considered for these equids, to minimise prolonged suffering. Moreover, owners’ education is recommended to guarantee minimal welfare standards to the working equids.

## 1. Introduction

With approximately 116 million horses, mules, and donkeys scattered around the globe [1], equines are one of the world’s most important working animals, second only to cattle [2]. Among these 116 million, one-quarter are present in the lowest-income countries [3]. Working equids are used for a variety of purposes, providing draught and carrying power for domestic and agricultural activities, as well as outputs such as manure and sometimes milk, meat, and hides [4,5]. These animals live their lives mainly working in harsh climatic conditions, without any rest or water supply [6,7] as they represent an essential source of income for their owners, who often live in poverty [8,9,10]. Notwithstanding their fundamental role within developing areas and their substantial socioeconomic contribution to their communities, published scientific evidence on their health and welfare conditions remains scant [11,12].

To date, researchers have mainly focused on identifying valid clinical parameters or protocols to easily assess the health and the welfare of working equids [13,14,15]. For instance, in 2008, the validity of several dehydration indicators was tested in working horses as dehydration is considered a severe welfare concern [14]. Reix and colleagues conducted a study in 2014 to identify the prevalence of clinical signs and limb conformation associated with lameness in working donkeys in Pakistan. In this study, it was reported that all the 102 assessed donkeys had gait abnormalities, with 5% having a non-weight-bearing limb [16]. Three years later, Salem et al. studied the prevalence and risk factors associated with colic in 350 working horses in Egypt. Horses with dental disease, displaying stereotypic behaviours, fed with ground corn, and that had received an anthelmintic in the previous 6 months were more likely to have had colic in the last 12 months [17].

The main conclusion of these studies is raising owners’ awareness of when the animal is sick and when they should be rested. Access to primary veterinary care and shelter areas is often limited by owners’ educational, financial, or logistical constraints [18]. Moreover, even if owners often consider their animals as family members, they use them, often to the point of exhaustion, as a means for covering their primary necessities [19,20]. As a consequence, these animals are used till exhaustion and death, which represents a remarkable welfare concern. For these reasons, most of the time, working horses, after a life of hard work, die in poor conditions, suffering till the very end. In this context, euthanasia, i.e., the production of a quiet, painless death for humane reasons [21], should be considered when necessary.

In these developing areas, non-governmental organisations, sanctuaries, and charity hospitals work to improve working equids welfare standards [22]. In these facilities, veterinarians often do not have diagnostic tools at their disposal, or can only use them to a limited extent, and must therefore rely exclusively on the animals’ physical signs and drug response. In Egypt, around the Cairo metropolis, working equids are numerous, also considering the touristic function operating around the Pyramid area. The Egypt Equine Aid (EEA) clinic is situated next to the Cairo area (Giza) and helps thousands of working equids every year, providing cures and hospitalisation. To date, studies analysing the general disease prevalence in Egyptian working equids are scant [20]. Furthermore, studies on clinical prognostic parameters have been performed especially for horses [23,24] but always in hospital settings and with a wide range of tools available. The prognostic value of clinical parameters has never been investigated in a population of working horses and donkeys in a primary care clinical setting, to the best of the authors’ knowledge.

Authors hypothesised that there are physical parameters that can help veterinarians promptly recognise animals in a life-threatening condition and with a higher probability of not surviving, in order to help in informing end-of-life decisions. Hence, this study aimed to describe the population of working equids around the Cairo area admitted to EEA from 2019 to 2022 and their disease prevalence. Moreover, possible associations between the physical parameters at admission and the outcome (i.e., discharged, euthanasia, or dead) were investigated.

## 2. Materials and Methods

### 2.1. Location and Equids Management

The study was conducted in Egypt at Egypt Equine Aid (EEA). EEA is an equine clinic situated in Giza (Cairo), Egypt. The EEA clinic routinely had a team of at least three veterinarians working simultaneously from 8 am to 5 pm. The veterinarians graduated in Egypt and had different levels and areas of specialisation (e.g., reproduction and orthopaedics). The EEA clinic was entirely supported and financed by donations and had the facilities to hospitalise up to 90 equids at the same time. Working equids in Egypt were mainly represented by native mixed breeds. During hospitalisation, animals were individually housed and managed according to their condition. Animals were fed hay three times a day and grain twice and had ad libitum water availability. On admission at EEA, a preliminary assessment of the animals was performed to determine the need for immediate examination and emergency treatment; otherwise, a standard protocol was applied. Animals often arrived at EEA walking for hours under the sun and sometimes also pulling the cart. On arrival, the clinic staff always provided them with water and shelter in the shade. Hence, while the owner was asked for the history, the animal had time to rest for a brief period before the clinical examination was performed. Generalities and clinical findings of the animals were collected on a paper-based medical record. In particular, the species, sex, and age of the animal were noted down. Most of the time, the age was based on the examination of the incisors of the animal [25]. Moreover, the anamnesis was reported by writing down what the owner said with his/her own words. Once a clinical examination was performed, the following physical findings were reported on the paper-based medical record: heart rate, rectal temperature, skin turgor, moisture of the mucous membrane, colour of the mucous membrane, capillary refill time (CRT), digital pulse, lungs, and abdominal auscultation.

Finally, a problem list (e.g., colic and degree of lameness) and a presumptive diagnosis were reported. Supplementary diagnostic testing (i.e., haemato-chemistry, ultrasounds, X-rays, and endoscopy) was performed on an individual basis but not usually recorded. A specific treatment plan was agreed among the veterinarians, indicating precisely the daily management and the therapeutic plan. At the end of the hospitalisation, the date, the outcome, and the length of the hospitalisation period were reported on the medical record.

The outcomes were the following:

1. Discharged: The animal has fully recovered and is ready to go home.
2. Discharged not fully recovered: The animal has partially recovered but the owner pushed for taking him home. The veterinarians agreed to it only knowing the animal would complete the recovery at home.
3. Euthanasia: Quiet and painless death performed with drugs by veterinarians, due to humane reasons [21].
4. Dead: Cessation of vital functions without human intervention or assistance.

### 2.2. Data Collection

In this study, all paper-based medical records of 1360 working equids admitted at EEA from 2019 to 2022 were included. In particular, the records included the date of admission, species, sex, age, anamnesis, heart rate, rectal temperature, skin turgor, mucous membrane moisture and colour, CRT, digital pulse, initial diagnosis, analgesics used, the outcome with its date, and the hospitalisation period length. Clinical cases that were euthanised or died right after arrival were included in the study. All the data registered at the admission were organised into an Excel data sheet (Microsoft Excel^®^, v16.0, Redmond, WA, USA) where each row represented a clinical case and columns reported the different variables (e.g., generalities, physical parameters, and outcome). A progressive identification number was given to each clinical case. If some information was missing, it was identified as missing data.

### 2.3. Data Handling

To perform the descriptive statistics of the dataset, some variables, originally reported as numeric, were categorised and other columns with additional variables were added ex post. Table 1 shows the final variables with their names and categories.

Considering what was reported in the anamnesis, two more variables were added. In particular, one variable reported the duration of the problem before admission, so the period between the owner’s identification of the signs and the arrival at EEA; and the other registered if the animal received any examination or treatment by a veterinarian, a farrier, or the owner before hospitalisation. Lastly, for the animals that received cures before admission, it was reported whether these treatments were considered appropriate or not by the EEA veterinarians.

The categories of the variables heart rate and rectal temperature took into account the age and the species [26,27,28,29] of the animals; more details on the reference ranges used for categorisation are reported in Tables S1 and S2.

The initial diagnosis was categorised considering 11 categories of disorders: wounds, orthopaedic problems, colic, infectious diseases, eye lesions, neurological problems, fracture, abscess, respiratory problems, starvation, and other. The category other included low-incidence diagnoses such as swelling, hematoma, congenital deformities, and sarcoids. Moreover, another column that considered the total number of diagnoses per animal was added. The hospitalisation period length, initially registered in days, was categorised considering ranges of periods.

To perform the univariable and multivariable ordinal logistic regression analyses, only horses and donkeys were retained, while mules were removed as they numbered only 19 (1.4%). Moreover, the cases which did not have an outcome were removed. Hence, the new dataset for regression analyses was composed of 1336 animals. To correctly perform the regression analyses, further categorisation of some variables was deemed necessary. In particular, categories that had an incidence below 5% were merged. This process was necessary also for the variable outcome, and the categories ‘discharged’ and ‘discharged not fully recovered’ were merged. Moreover, for the variables colour of the mucous membrane and capillary refill time (CRT), a further categorisation into two categories (i.e., physiological/pathological) was necessary to better fit the statistical model. Details on this further categorisation are reported in Table S3.

### 2.4. Statistical Analysis

Descriptive statistics were performed on all variables shown in Table 1 and were obtained using Statulor^®^ (https://statulator.com/, accessed on 10 September 2023). Descriptive statistics on the number of cases were also performed divided per year. Descriptive statistics were also performed on the single diseases (i.e., piroplasmosis, tetanus, habronema, and vesicular stomatitis) related to the category ’infectious disease’, while for the category orthopaedic problems, descriptive statistics were also performed according to the age of the animal. Results were reported as frequency (n) and percentage (%) of each category and the missing data. For the variable hospitalisation period length, descriptive statistics were performed also on the numeric data (i.e., days) and reported as the median, minimum, maximum, and first and third quartiles (Q1–Q3).

Text mining analysis was performed on the variable anamnesis in order to highlight the most frequent words used by the owners to describe the history of the animal. A separate Excel sheet with two columns, namely progressive ID and anamnesis text, was prepared. TM analysis was performed in the R environment using a combination of functions in the packages ‘tm’, ‘SnowballC’, ‘ggplot2’, ‘dplyr’, and ‘tidyverse’. Pre-processing steps were conducted as described in the literature [30]. To best extrapolate the most frequent words, the stopwords ‘owner’, ‘horse’, ‘donkey’, ‘got’, ‘day’, ‘ago’, ‘another’, ‘start’, ‘sign’, ‘anamnesis’, ‘since’, and ‘from’, which are common and of minor interest, were removed. Text stemming, which reduces the words to their roots, was not performed. The term frequency-inverse document frequency technique (TFIDF) was used to weigh the words [31]. The first 15 words were represented as a histogram.

Univariable and multivariable ordinal logistic regression was performed to identify possible associations between factors (i.e., species, sex, heart rate, rectal temperature, mucous membrane moisture, colour of the mucous membrane, capillary refill time (CRT), and digital pulse) and the outcome. The ordinal logistic regression was performed with the outcome as the dependent variable, considering the categories discharged (the categories discharged and discharged not fully recovered were merged), euthanised, and dead, respectively ordered according to their gravity. The proportional odds assumption was tested for its validity, observing the differences between the coefficient of the stratified binomial models (all differences < 2.00 in the multivariable model) [32]. The factors (independent variables) that had more than 25% of missing data were not considered for regression analyses. Moreover, the factor hospitalisation period length was not included as it does not match the aim of the study (i.e., find some parameters at admission associated with death). Dependence, tested with the X^2^ test, was detected between the factors of skin turgor and CRT (*p* < 0.001). It was decided to maintain in the model the factor CRT, which was considered to be more accurate in the literature [33]. The factors that showed a *p*-value ≤ 0.250 were considered for inclusion in the stepwise multiple regression. The model was built using a backward manual stepwise method in which the least significant factors were dropped until all factors were associated with the dependent variable at *p* < 0.05. Both the univariable and multivariable models were performed in the R environment using functions of the packages ‘foreign’, ‘MASS’, ‘Hmisc’, ‘reshape2’, ‘ordinal’, ‘lmtest’, and ‘car’. The results were reported as estimate ± standard error (SE), odds ratios (ORs), confidence interval (CI 5–95%), and Wald test *p*-values. The significance threshold was set at a *p*-value ≤ 0.05.

## 3. Results

### 3.1. Descriptive Statistics

The number of clinical cases divided per year and outcome are reported in Table 2. Table 3 shows the descriptive statistics of the variables species, sex, age, and hospitalisation period length of the population. The majority of clinical cases were horses (66.6%), mainly stallions (68.7%), and aged from 1 to 15 years (74.8%). In 34% of the cases, age was not reported (missing value). Most of the cases were hospitalised for less than a month (70.5%), but there was also a relevant part that was hospitalised for more than 2 months (10.1%). The outcome was mainly positive, with 71.9% of animals discharged, while the total percentage of non-surviving animals, considering both death and euthanised ones, was 28.1%. Euthanised animals were hospitalised for a median of 2 days (min–ax = 0–212; Q1–Q3 = 0–10), while equids that died on their own were at EEA for a median of 3 days (min–max = 0–72; Q1–Q3 = 1–11).

Considering the variable anamnesis, in 37.6% of cases, the owner specified the duration of the problem. Within these cases, one-quarter arrived at EEA almost in a short period after the main problem occurred (within 48 h), another quarter arrived after 2–3 days, and 20.9% after 4–7 days. Finally, 12.7% of owners brought the animal to the clinic 10–15 days after the manifestation of the main problem, and 13.3% after 20 days or more. The majority of owners brought the animal directly to EEA (87.4%), while the remaining part of owners visited another veterinarian (4.8%) or farrier (2.1%) or administered therapies themselves (5.7%) before bringing the animal to the clinic. Furthermore, most of the animals (72.5%) that received cures before arriving at EEA (129 equids) were subjected to treatments considered inappropriate or misguided by the EEA veterinarians. A list of these inappropriate treatments with their relative description and their frequency and percentage are summarised in Table 4.

The distribution of physical parameters of heart rate, rectal temperature, skin turgor, mucous membrane moisture and colour, CRT, and digital pulse taken at admission are reported in Table 5. Almost 71% of the animals had an increased heart rate at admission, while the rectal temperature was altered in 37.5% of cases. Considering the mucous membrane, almost 10% of the animals arrived with a sticky mucous membrane, and the most frequent pathological colour was pale.

One main diagnosis was formulated for 89.9% of clinical cases, while 9.8% received two diagnoses, and 0.2% three different diagnoses. The frequency and percentage of each category of initial diagnosis are reported in Table 6. The most reported diagnoses were wounds, orthopaedic problems, and colic. Almost half of the animals (45.0%) who suffered from orthopaedic problems were less than 5 years old. Among the infectious diseases, the most common were tetanus (61%) and piroplasmosis (22%). Neurological and respiratory problems were the least reported.

Only in 7% of cases was the treatment with analgesics considered unnecessary. A further 6% received no analgesics because they were immediately euthanised at arrival for a condition of extreme suffering and poor prognosis (e.g., fracture of the cannon bone). The remaining animals received treatment with NSAIDs. In particular, 45% received Phenylbutazone and 41.9% received Flunixin meglumine.

### 3.2. Text mining on Anamnesis

**Figure 1:**
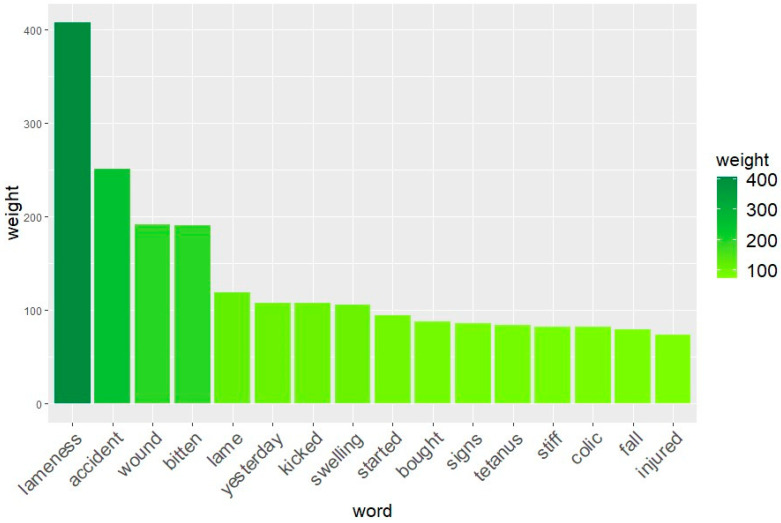
Histogram of the 15 most relevant words revealed with TM analysis and their relative weights, used by the owners to describe the anamnesis of the working equids brought to EEA from 2019 to 2022.

The TM analysis performed on the anamnesis text revealed the 15 most frequent words, summarised in Figure 1. This analysis revealed the most common words used by the owners to describe the history of the animal and its main problem. The four most frequent words were ‘lameness’, ‘accident’, ‘wound’, and ‘bitten’ with TFIDF weights of 407.1, 251.0, and 191.6 for the las two, respectively.

### 3.3. Univariable and Multivariable Regression Analyses

**Figure 2:**
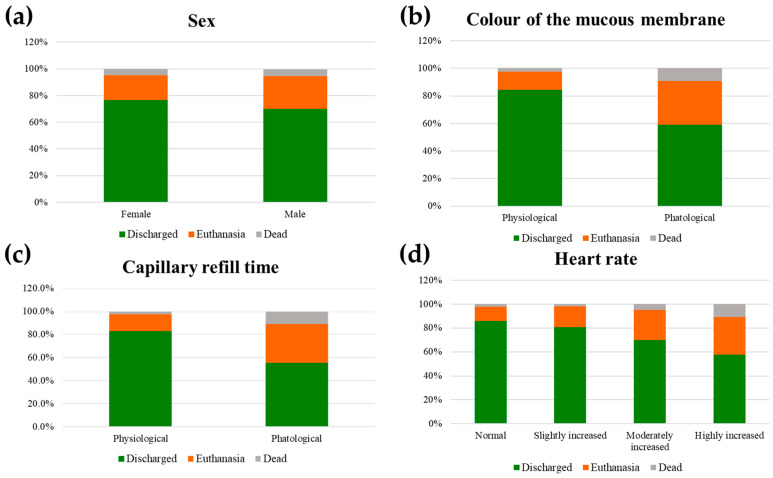
Bar charts representing the probability of each possible outcome (green = discharged; orange = euthanasia; grey = dead) associated with sex (**a**), colour of the mucous membrane (**b**), capillary refill time—CRT (**c**), heart rate (**d**).

Table 7 shows the *p* values of the factors considered in the univariable ordinal regression model with the dependent variable outcome. For the factors that resulted significant, also the estimate ± SE, OR, and CI (5–95%) were reported. Figure 2 shows the bar charts of the factors that resulted significant (*p*-value < 0.05), namely sex, heart rate, colour of the mucous membrane, and CRT.

Being male increased the odds of a non-survival outcome by 1.30 times compared to being female. A highly elevated heart rate increased the odds of a non-survival outcome by 1.95 times compared with a physiological heart rate. Pathological mucous membrane colour increased the odds of euthanasia or death by almost two times compared with physiological colour. In addition, the odds of a non-survival outcome were 1.8 times higher with a pathological CRT than with a physiological CRT.

Table 8 shows the significant factors included in the final multivariable ordinal model for the dependent variable outcome. In particular, male equids, with a pathological mucous membrane and pathological CRT, resulted to have a higher likelihood of a non-survival outcome (i.e., euthanasia or death).

## 4. Discussion

This study aimed to describe the main features of the population of working equids hospitalised at the Egypt Equine Aid (EEA) clinic from 2019 to 2022, exploring which are the main causes of hospitalisation according to the main diagnosis and owner perception. Furthermore, the study aimed to find any possible physical parameter registered at admission associated with an increased likelihood of a non-survival outcome, namely euthanasia or death. These parameters, when present concurrently, could serve as a screening to highlight the most critical patients. The study revealed that the animals hospitalised at EEA were mainly male horses and donkeys suffering from wounds, orthopaedic problems, and colics. The owners brought them to the clinic principally because of car accidents or due to lameness or bites from other animals. Male subjects with a pathological mucous membrane colour and pathological CRT were associated with a non-survival outcome in this population. These parameters can be used as prognostic factors, especially in a clinical context (i.e., charity hospitals) where there is no possibility of using other supportive diagnostic tools. In this case, animals should be evaluated carefully, managed as critical cases, and earlier euthanasia should be considered.

In our population, the number of hospitalised equids slightly increased over the years and, with this, also the number and percentage of euthanised and dead horses. The exception is represented by the year 2020 in which, due to the COVID-19 pandemic, the number of equids admitted to the hospital was reduced (only 6.2% of the total population, *n* = 1360). It is worth emphasising that the clinic in that year acted as a shelter for the majority of cases, hosting horses and donkeys of owners who could no longer keep them due to the COVID-19 pandemic. On the other hand, with the slowdown in work activities and road traffic, the incidence of disease and therefore hospitalisation may have reduced. These data, such different for 2020, justify why the variable year was not included in the regression analysis.

Working equids admitted at EEA were mainly horses, but donkeys were also present, being one-third of the clinical cases registered. This could be due to two main reasons; firstly, donkeys are more resilient and stoic than horses [34], and secondly, they tend to hide the expressions and behaviour of pain more than horses [27]. Either one of these two reasons or a combination of both could have meant that donkeys who arrived at EEA were less in percentage than horses, potentially not representing the real frequencies of working equids in Egypt [35]. Moreover, horses are considered to have a higher economic value than donkeys [20], and for this reason, owners may have been more alert and motivated to bring their horses to the clinic.

Stallions represented more than two-thirds of animals that arrived at EEA. Moreover, the majority of animals were from 1 to 15 years old, while very old animals (more than 20 years) were very rare. Most of the working equids are, in fact, not castrated due to owners’ economic restrictions and unwillingness to stop the animal for a few weeks. This is in line with what was reported by Salem and colleagues in 2017 [17], who found only 2 (0.6%) geldings analysing a sample of 350 working horses in Egypt. This point represents one of the main concerns of working equids; having young stallions, with low levels of training, stabled in not-well-secured facilities may elicit an increase in social competition and thus aggression. Indeed, they tend to attack both people and other animals/horses. As also reported in this study, this resulted in many wounds and injuries, such as neck and wither bites, caused by fighting with other stallions. Moreover, these equids usually have a low level of training that could bring out fear and avoidance behaviours in a chaotic environment such as Cairo city. These behaviours often lead to improper handling and vice versa, which can become dangerous for people as well as for animals [36,37]. Finally, two considerations must be made about the age of these animals. Working equids often start working before their musculoskeletal system has fully developed, which happens roughly at the age of 2 years in racing horses [38]. Moreover, equids continue to develop until around the age of 5 years [19,37], and this should be taken into account when deciding their workload [37]. Nonetheless, a large proportion of animals arriving at EEA due to lameness were young equids (less than 5 years old); thus, it could be assumed they started their work too early and were not fully able to support the weight of the cart, causing orthopaedical problems and back pain. It is to be considered that animals that are subjected to excessive work while too young will usually have a much-reduced working life [37]. Age is an important factor when talking about the welfare of working equids; and as suggested by McLeod, the more appropriate age range for working is between 4 and 12 years old [39]. Older equids were rare in this study, probably not because they were withdrawn from work at the appropriate age but because they died early due to overuse or were abandoned or neglected when they were deemed no longer able to sustain the required workload [40]. All the factors mentioned above need to be considered by the owners as they not only affect the general welfare status of working equids but also the owner’s profits. An educational campaign on these aspects should therefore be carried out to make all owners and those who care for these equids aware that only by acting on these characteristics (castration, age, and training) can the standard of health and welfare of these equids greatly improve [37]. It is well known that equid owners, when interviewed, often refer to their working animals as family members [19]. Hence, it is difficult to understand why they are not aware of the low levels of welfare to which their horses are subjected. Therefore, it is essential to dig deeper into the cultural context and social structure of these people to better understand the contradiction between what they believe and how they actually manage their animals. Understanding this could be the key point to make educational initiatives more effective in the future [41].

With the word analysis conducted by text mining, it was revealed that among the words most frequently reported by the owners when telling the history of the animal (anamnesis), there was the word ‘accident’. This is, indeed, another important point regarding the health and quality of life of these animals. In crowded cities such as Cairo, animals often work next to cars and motorbikes. The possibility of traffic accidents between these animals and vehicles is unfortunately still a very common reality. Most of the time, it is the equid that gets the worst of it, suffering serious life-threatening injuries such as fractures and deep wounds. Hence, the World Organisation for Animal Health (WOAH) suggested that ‘Particularly in urban areas, the transport or other responsible agency may have legislative authority in dealing with traffic circulation and have a role to play in ensuring a safe environment for working equids as well as other road users’ [37].

It is interesting to note that no words related to pain have been retrieved with the text mining analysis. This means that owners usually refer animals to the EEA clinic mainly because of macroscopic problems, such as a wound or lameness, but when the anamnesis is asked, only a few people highlight that the horse/donkey is in pain. Pain recognition and assessment are difficult in prey species such as equids and it is well recognised only by trained and expert people [42]. Nonetheless, easy tools to assess pain have been developed in the last years [43,44] and should be shared among owners. This can potentially be of help to owners, who may be able to recognise pain early and bring animals earlier and with less severe diseases. An earlier admission might increase the probability of recovery.

The diseases found to be the most frequent in this study confirm the main causes above discussed. Wounds and orthopaedic problems represented the majority of disorders in accordance with the main management problems, namely the early age at which the equids start to work, the aggression among stallions, and frequent road accidents.

Consideration must be given to colics and infectious diseases. Colics are the most frequent disease among horses [45], so it is not surprising that colics are also represented in this study population. The main typologies reported in working equids are intestinal obstruction due to rubbish ingestion, such as polyethylene bags or nylon clothes [46], and thromboembolism due to strongylosis [47]. Although these are the most common aetiologies, owners often think that colics are caused by obstructions of the urinary tract. That is why, as also reported in this study, colic is often treated inappropriately with diuretics [17]. This treatment is counterproductive, as it only worsens the already compromised haemodynamic state of a colicky horse, worsening the course of the disease and prognosis of the horse. Encouraging owners to prevent working equids from grazing at waste disposal sites and informing them about the fact that urinary obstructions are unlikely to happen in horses and donkeys [48] are the priorities to be communicated to owners to reduce colic incidence in Egypt and all the developing countries [49].

With regard to infectious diseases, the most frequent is certainly tetanus. This trend is mainly related to the incidence of wounds and the economic reality in Egypt, which does not make tetanus prophylaxis a large-scale viable option [49]. Even if some alternative strategies could be applied (e.g., prevention of wounds, prompt wound cleaning, hyperimmune serum), vaccination prophylaxis is of the utmost importance and should be encouraged, also because of the pain that tetanus causes and the subsequent suffering of death. In addition, once established, tetanus is very difficult and expensive to treat and has a high mortality rate [50].

One of the aims of the study was to investigate the prognostic value of physical parameters assessed on admission. The physical parameters found associated in the univariable regression analysis, namely heart rate, colour of the mucous membrane, and CRT, are easy to assess and monitor and, for this reason, can help in the screening of a clinical case. In settings like the EEA that rely solely on donations and cannot afford the routine use of diagnostic tools and veterinary personnel on night shifts, promptly recognising which animals are most likely to die or that will need euthanasia is crucial. The parameters found can act as prognostic factors upon admission and inform the decision of veterinarians who work with clinical settings similar to EEA to suggest euthanasia, avoiding further suffering for the animal. Regardless, euthanasia must be carefully weighed and discussed with the owner. Often, the literature specifies that the final decision must be made by considering whether the animal’s life is worth living [51], looking at the pain the animal is suffering [52], and the type of pathology [53]. Our study tried to identify some basic parameters that far from being exhaustive could help to inform this decision, in conditions where often no more than a physical examination can be performed and with limited treatment and assistance availability. In this context, these physical parameters may have even more relevance because no other diagnostic tools are available to better characterise the clinical picture and prognosis. Heart rate, colour of the mucous membrane, and CRT are very well-acknowledged prognostic factors in the equine literature [54,55]. In this study, their prognostic value has been documented and confirmed also in this equid population and clinical settings, as expected. Some comparisons can be made with other equine populations with regard to these parameters, in particular endurance horses. In endurance competitions, these findings are signs of severe metabolic and haemodynamic impairment related to the great effort of the race [56]. In particular, the exhaustion syndrome, a severe complication of these pathological events, appears to have similarities to the conditions found in the observed population. Dehydration and electrolyte consumption were the major factors that determine the signs of exhaustion and could be severe enough to cause hypovolemic and circulatory shock, resulting in a cascade of irreversible events [57]. On the other hand, many different factors have to be considered when comparing endurance horses to working horses and donkeys, such as the breed, which is selected on purpose for this kind of competition, the management of a sport horse, and the basal health status prior to the competition. In working equids, acute dehydration due to exercise and thermal stress was assumed to be superimposed on chronic dehydration resulting from poor nutritional and water management and possibly anaemia, all contributing to impair welfare status and working capacity [13]. Hence, the parameters of heart rate, colour of the mucous membrane, and CRT, which represent the haemodynamic state of the animal and were found related to non-survival outcome, could be paramount in detecting this status. In this context, these physical parameters have added relevance because they are often the only ones that the veterinarian can rely on as no other diagnostic tools are available to better characterise the clinical picture and thus the severity and prognosis. However, it is also important to highlight that these parameters alone are not exhaustive. Nevertheless, other diseases and painful conditions affected horses and donkeys in this population. Highly increased heart rate has already been related to pain and haemodynamic imbalances [58]. Moreover, heart rate resulted as a valuable prognosis predictor during colic [55] and it is included in the parameters needed for the diagnosis of Systemic Inflammatory Response Syndrome (SIRS) [54]. In the same study, the abnormal colour of the mucous membrane was retained in the model for the prediction of severe SIRS, that was correlated with poor prognosis in horses with gastrointestinal diseases [54]. Nevertheless, many nonspecific factors can affect heart rate, such as temperament and hypovolemia, and the association between tachycardia and pain is not always so direct [59]. Colour of the mucous membrane and capillary refill time are easy to assess and objective [60] and, therefore, are factors that should be monitored constantly and can be an alarm to observe the patient further. Having these animals more under control could potentially help in planning a timely euthanasia, consequently reducing the percentage of equids that were dying alone potentially suffering (which was 5.1% in this population).

It is also worth pointing out the average number of days spent in the clinic by the animals before they are put to sleep or die. Horses lived for 2–3 days (median values) before being euthanised or dying. During this period, pain was carefully controlled. Nevertheless, this amount of time, though with exceptions, could be shortened as much as possible to avoid prolonged suffering. In addition, the consideration of early euthanasia in charity-based clinics should also be considered to better manage the economic resources.

The multivariable analysis showed that male equids with a pathological mucous membrane colour and CRT were associated with a non-survival outcome. The animals with these characteristics were found to be the most at risk in this population; therefore, the decision to euthanise must be considered very carefully. The male sex was associated with a non-survival outcome probably because male horses and donkeys are considered stronger and their workload is higher than females, leading to an increased possibility of exhaustion.

Although this study achieved important results and described the situation of working equids in the area around Cairo, limitations are present. The retrospective nature of the study did not always allow the initial or presumptive diagnoses described to be confirmed by means of supplementary diagnostic testing. Moreover, because of the nature of the study, some important parameters such as age had a high amount of missing data, and for this they could, unfortunately, not be included in the regression analysis. Furthermore, no monitoring parameters taken during hospitalisation were included in the analysis. In addition, some physical parameters taken at arrival could be partially affected or worsened by the effort that the animals had to make to arrive at the clinic and the short rest period prior to the clinical examination. Finally, the regression analysis was based on simple physical parameters and the inclusion of blood sample testing and collateral diagnosis exams could have provided more information. However, bloodwork and other exams cannot be routinely performed in charity hospitals such as the EEA, and for this reason, they could not be included in the analysis. Lastly, it is worth highlighting that the statistical approach could have been affected by the suboptimal sample size and, in particular, by the limited representation of ‘dead’ animals, which constituted only 5.1%. The interpretation of the results of the ordinal regression analyses should therefore be taken with care, and it should be remembered that it only refers to the analysed population. Nevertheless, even if the proportional odds assumption was verified, indicating that the statistical model is fit for the dataset, the indexes could have been better. In future studies, it is therefore desirable to have a larger sample size and a better balance between outcome categories (i.e., discharged, euthanasia, and dead) to allow for a more robust interpretation of the results.

Notwithstanding these limitations, this study is a field-based study and our data have never been presented before; this study filled, therefore, the gap in knowledge on the general frequency of the diseases of Cairo working equids admitted at a charity hospital, providing pivotal information. It should be considered as a starting point to gain a better understanding of the main problems of working horses and donkeys around the Cairo area and how to manage them.

## 5. Conclusions

The results of this retrospective study on working equids admitted at the EEA clinic from 2019 to 2022 showed that the population was mainly stallion horses, aged mainly from 1 to 15 years. They were hospitalised mainly due to car- or harness-related wounds, orthopaedic problems, colics, or infectious diseases. Anamnestic data showed that owners sometimes did not manage their animals properly, performing treatments that were considered inappropriate by the examining veterinarians. Among the physical parameters assessed at arrival, heart rate, colour of the mucous membrane, and CRT were associated in the univariable ordinal regression with euthanasia and death in this population. These physical parameters could serve as a screening for veterinarians working in similar clinical settings to predict non-survival and consider early end-of-life decision making. Considering the multivariable ordinal regression, male equids with a pathological colour of the mucous membrane and pathological CRT at arrival were associated with a non-survival outcome. For this category, euthanasia must be pondered carefully. The decision to euthanise must, however, be weighed up by deeply analysing the individual case and the overall clinical picture together with the availability of proper assistance and treatment.

## Figures and Tables

**Table 1:** Name and categories of the categorical variables included in a retrospective study on working equids that arrived at Egypt Equine Aid from 2019 to 2022.

Variable Name	Categories
Species	Horse, donkey, mule
Sex	Stallion, gelding, female
Age	<1 month, <1 year, 1–5 years, 5–15 years, 15–20 years, >20 years
Anamnesis	Reporting what the owner said, with his/her words
Duration of signs before admission	Within 48 h, after 2–3 days, after 4–9 days, after 10–15 days, >20 days
Previous treatments	Directly at EEA, examined by another vet, examined from a farrier, owner administered therapies
Treatments before hospitalisation	Faulty injection, diuretic, firing, inappropriate shoes or over-trimming, suture, other management (e.g., no drink for >24 h, clean wounds with chemical cleaners), seaton, castration
Heart rate	Normal, slightly increased, moderately increased, highly increased
Rectal temperature	Normal, slightly increased, moderately increased, highly abnormal (i.e., highly increased or highly decreased)
Skin turgor	Normal, reduced
Mucous membrane moisture	Moist, sticky
Colour of the mucous membrane	Pink, pale, congested, cyanotic, icteric, toxic line
Capillary Refill Time (CRT)	Normal (2 s), slightly increased (3 s), highly increased (≥4 s)
Digital pulse	Not palpable, on one leg, on two legs, palpable on all four legs
Initial diagnosis	Wound, orthopaedic problems, colic, infectious diseases (i.e., piroplasmosis, tetanus, habronema, and vesicular stomatitis), eye lesions, neurological problems, fracture, abscess, respiratory problems, starvation, other (i.e., swelling, hematoma, congenital deformities, and sarcoids)
Number of diagnoses	One, two, three or more
Analgesics	None, not administered due to immediate euthanasia, Flunixin Meglumine, Phenylbutazone
Outcome	Discharged, discharged not fully recovered, euthanasia, dead
Hospitalisation period length	<1 week, 1 week–1 month, 1–2 months, >2 months

**Table 2:** Details per year of the number of equids and the relative outcome of the population of equids analysed in a retrospective study on working equids that arrived at EEA from 2019 to 2022. Results are reported as frequency (*n*) and percentage (%). In the column number of cases, the percentage reported indicates the value of cases of that year compared with the total cases (*n* = 1360). The missing values are indicated separately.

Year	Number of Cases	Discharged	Discharged Not Fully Recovered	Euthanasia	Dead
2019	369 (27.1%)	244 (66.1%)	35 (9.5%)	76 (20.6%)	14 (3.8%)
2020 ^1^	84 (6.2%)	64 (76.2%)	2 (2.4%)	5 (6%)	12 (14.3%)
2021	420 (30.9%)	284 (67.6%)	16 (3.8%)	102 (24.3%)	18 (4.3%)
2022 ^1^	487 (35.8%)	316 (64.9%)	13 (2.6%)	129 (26.5%)	25 (5.1%)
Total ^1^	1360 (100%)	908 (66.7%)	66 (4.9%)	312 (22.9%)	69 (5.1%)

^1^ Missing data: Data were missing for some variables, and the denominator for percentage calculations is based on the number of horses for which data were available. Data on the outcome were missing for year 2020 (missing 1, 1.2%) and year 2022 (missing = 4, 0.8%), total (missing = 5, 0.4%).

**Table 3:** Details (species, sex, age, and hospitalisation period length) of the population of equids analysed in a retrospective study on working equids that arrived at EEA from 2019 to 2022. Results are reported as frequency (*n*) and percentage (%).

Variable Name	Categories	Frequency (*n*)	Percentage
Species	Horse	892	65.6%
	Donkey	449	33.0%
	Mule	19	1.4%
Sex (*n* = 1356) ^1^	Stallion	931	68.7%
	Gelding	18	1.3%
	Female	407	30.0%
Age (*n* = 898) ^1^	<1 month	16	1.8%
	<1 year	86	9.6%
	1–5 years	336	37.4%
	5–15 years	336	37.4%
	15–20 years	80	8.9%
	>20 years	44	4.9%
Hospitalisation period length (*n* = 1336) ^1^	<1 week	371	27.8%
	1 week–1 month	570	42.7%
	1–2 months	261	19.5%

^1^ Missing data: Data were missing for some variables, and the denominator for percentage calculations is based on the number of horses for which data were available. The total number of horses with available data is indicated next to each variable name. Data were missing for sex (missing = 4, 0.3%); age (missing = 462, 34.0%); and hospitalisation period length (missing = 5, 0.4%).

**Table 4:** Name, description, frequency (*n*), and percentage (%) of the inappropriate treatments to the animals considered in a retrospective study on working equids that arrived at EEA from 2019 to 2022. The total number of equids who received inappropriate treatments is 129.

Inappropriate Treatment	Description	Frequency (*n*)	%
Faulty injection	Intramuscular or intravenous injections performed in non-sterile conditions, with dirty needles. In most cases, they lead to swellings, neck/rump abscesses, or phlebitis.	29	22.5%
Diuretic	Diuretic drugs are commonly misused during colic, worsening the state of hydration already compromised by the disease.	24	18.6%
Firing	Causing one or more burn wounds is a practice misused with the idea of facilitating the healing of certain disorders such as laminitis, lameness, diarrhoea, and others.	18	14.0%
Inappropriate shoes or over-trimming	The farrier cuts the nail too much or applies shoes unsuitable for the animal.	16	12.4%
Suture	Stitches placed by the owner or inexperienced persons, in non-sterile conditions and often with unsuitable threads (e.g., suturing the animal using its hair as thread). This causes the sutures to break and the wound to become infected.	15	11.6%
Other management	Incorrect management by the owner. Examples are giving the animal many kilos of grain, not letting the animal drink for more than 24 h, and applying chemical cleaners and/or alcohol to wounds.	14	10.9%
Seaton	Leaving a ‘stitch’ made with a rope on one or different parts of the animal’s body for a few days/weeks with the same intent as firing.	10	7.8%
Castration (performed by inexperienced people)	This surgical procedure should only be performed by veterinarians under sterile conditions and with the animal properly sedated/anesthetised. Improvisation of this surgery by inexperienced persons leads to complications such as bleeding and evisceration.	3	2.3%

**Table 5:** Details on physical parameters of heart rate, rectal temperature, skin turgor, mucous membrane moisture and colour, CRT, and digital pulse of the population analysed in a retrospective study on working equids that arrived at EEA from 2019 to 2022. Results are reported as frequency (*n*) and percentage (%). The total number of cases and the missing values are indicated separately.

Variable Name	Categories	Frequency (*n*)	Percentage
Heart Rate (*n* = 1278) ^1^	Normal	373	29.2%
	Slightly increased	327	25.6%
	Moderately increased	245	19.2%
	Highly increased	333	26.1%
Rectal temperature (*n* = 1251) ^1^	Normal	782	62.5%
	Slightly increased	243	19.4%
	Moderately increased	117	9.4%
	Highly abnormal	109	8.7%
Skin turgor (*n* = 1267) ^1^	Normal	939	74.1%
	Reduced	328	25.9%
Mucous membrane moisture (*n* = 1248) ^1^	Moist	1124	90.1%
	Sticky	124	9.9%
Colour of the mucous membrane (*n* = 1249) ^1^	Pink	787	63.0%
	Pale	298	23.9%
	Congested	109	8.7%
	Icteric	33	2.6%
	Toxic line	17	1.4%
	Cyanotic	5	0.4%
CRT (*n* = 1230) ^1^	Normal (2 s)	878	71.4%
	Slightly increased (3 s)	294	23.9%
	Highly increased (≥4 s)	58	4.7%
Digital pulse (*n* = 1034) ^1^	Not palpable	914	88.4%
	On one leg	38	3.7%
	On 2 legs	45	4.4%
	Palpable on all 4 legs	37	3.6%

^1^ Missing data: Data were missing for all variables, and the denominator for percentage calculations is based on the number of horses for which data were available. The total number of horses with available data is indicated next to each variable name. Data were missing for heart rate (missing = 82, 6%); rectal temperature (missing = 109, 8.0%); skin turgor (missing = 93, 6.8%); mucous membrane moisture (missing = 112, 8.2%); colour of the mucous membrane (missing = 111, 8.2%); CRT (missing = 130, 9.6%); and digital pulse (missing = 326, 24%).

**Table 6:** Details of different initial diagnoses of the population analysed in a retrospective study on working equids that arrived at EEA from 2019 to 2022. Results are reported as frequency (*n*) and percentage (%). The total number of cases and the missing values are indicated separately.

Type of Diagnosis	Frequency (*n*)	Percentage *
Wound	393	28.9%
Orthopaedic problems	373	27.4%
Other	205	15.1%
Colic	116	8.5%
Infectious disease	100	7.4%
Fracture	81	6.0%
Abscess	72	5.3%
Starvation	56	4.1%
Eye lesions	37	2.7%
Neurological problem	34	2.5%
Respiratory problem	24	1.8%
Missing data	8	0.6%

* Total percentage: Note that as some animals had more than one initial diagnosis, the total percentage is greater than 100%.

**Table 7:** Univariable ordinal logistic regression model. Wald test *p*-values are reported for all the factors included in the analysis. For the factors that resulted significant, data are presented as estimate ± standard error (SE), odds ratio (OR), confidence interval (CI), and categories’ *p*-values. Ref = reference category.

Variable	Estimate ± SE	OR	CI (5–95%)	*p*-Value
**Species**				0.813
**Sex**				0.032
Female	Ref			
Male	0.287 ± 0.134	1.333	1.070–1.664	0.032
**Heart Rate**				<0.001
Normal	Ref			
Slightly increased	0.316 ± 0.176	1.372	1.027–1.834	0.072
Moderately increased	0.569 ± 0.189	1.767	1.294–2.412	0.003
Highly increased	0.668 ± 0.176	1.951	1.461–2.610	0.0001
**Rectal temperature**				0.597
**Mucous membrane moisture**				0.427
**Colour of the mucous membrane**				<0.001
Physiological	Ref			
Pathological	0.644 ± 0.137	1.904	1.521–2.384	0.0001
**Capillary Refill Time—CRT (s)**				<0.001
Physiological	Ref			
Pathological	0.594 ± 0.146	1.811	1.422–2.303	<0.001
**Digital pulse**				0.376

**Table 8:** Final multivariable ordinal regression model for the dependent variable ‘outcome’ ordered according to its severity (i.e., discharged, euthanised, and dead). Data are presented as estimate ± standard error (S.E.), odds ratio (OR), confidence interval (CI), and *p*-value. Ref = reference category. The model *p*-value calculated with the Wald test was <0.001.

Variable	Estimate ± SE	OR	CI (5–95%)	*p*-Value
**Sex**				0.050
Female	Ref			
Male	0.283 ± 0.148	1.327	1.042–1.696	0.050
**Colour of the mucous membrane**				<0.001
Physiological	Ref			
Pathological	0.541 ± 0.150	1.718	1.343–2.198	<0.001
**Capillary Refill Time—CRT (s)**				0.020
Physiological	Ref			
Pathological	0.348 ± 0.159	1.416	1.089–1.840	0.020

## Data Availability

Individual raw data collected are available on request from the corresponding author.

## Supplementary Materials

The following supporting information can be downloaded at https://www.mdpi.com/article/10.3390/ani14050817/s1, Table S1: Categorisation of heart rate (beats per minute, bpm) according to the age and the species of the animals [26,27,28,29] in a retrospective study on working equids that arrived at Egypt Equine Aid from 2019 to 2022; Table S2: Categorisation of temperature (°C) according to the age and the species of the animals [26,27,28,29] in a retrospective study on working equids that arrived at Egypt Equine Aid from 2019 to 2022; Table S3: Recategorisation needed for ordinal regression analyses.
