# Supplementary material for: A Retrospective Study on the Status of Working Equids Admitted to an Equine Clinic in Cairo: Disease Prevalence and Associations between Physical Parameters and Outcome
Source: Animals (Basel). 2024 Mar 6;14(5):817. doi: 10.3390/ani14050817 (PMC10930472; doi:10.3390/ani14050817)
Supplement: Supplementary file 1 [file animals-14-00817-s001.zip › Supplementary/Table S1.docx]

**Table S1.** Categorisation of Heart rate (beats per minute -bpm) according to the age and the species of the animals [26-29] in a retrospective study on working equids arrived at Egypt Equine Aid from 2019 to 2022.

| **Categories** | **Adult horse** [26] | **Adult donkey** [27] | **Foal horse 0-30 days** [28] | **Foal horse 1-6 months** [28] | **Foal donkey 0-30 days** [29] |
| --- | --- | --- | --- | --- | --- |
| Normal | 24-44 | 32-52 | 80-100 | 45-89 | 80-120 |
| Slightly increased | 45-52 | 53-60 | 100-115 | 90-115 | 120-140 |
| Moderately increased | 53-60 | 61-68 | 115-160 | 115-140 | 140-180 |
| Severely increased | >60 | >68 | >160 | >140 | >180 |
