# Supplementary material for: A Retrospective Study on the Status of Working Equids Admitted to an Equine Clinic in Cairo: Disease Prevalence and Associations between Physical Parameters and Outcome
Source: Animals (Basel). 2024 Mar 6;14(5):817. doi: 10.3390/ani14050817 (PMC10930472; doi:10.3390/ani14050817)
Supplement: Supplementary file 1 [file animals-14-00817-s001.zip › Supplementary/Table S2.docx]

**Table S2.** Categorisation of temperature (°C) according to the age and the species of the animals [26-29] in a retrospective study on working equids arrived at Egypt Equine Aid from 2019 to 2022.

| **Categories** | **Adult horse** [26] | **Adult donkey** [27] | **Foal horse 0-30 days** [28] | **Foal donkey** [29] |
| --- | --- | --- | --- | --- |
| Normal | 36.9-38.5 | 35.7-38.0 | 37.2-39.2 | 37.5-38.5 |
| Slightly increased | 38.6-39.0 | 38.1-38.5 | 39.3-39.7 | 38.6- 39.0 |
| Moderately increased | 39.1-39.5 | 38.6-39.0 | 39.8-40.2 | 39.1-39.5 |
| Highly abnormal | <36.8->39.5 | <35.3->39.0 | <37.1->40.2 | <37.4->39.5 |
