# Supplementary material for: A Retrospective Study on the Status of Working Equids Admitted to an Equine Clinic in Cairo: Disease Prevalence and Associations between Physical Parameters and Outcome
Source: Animals (Basel). 2024 Mar 6;14(5):817. doi: 10.3390/ani14050817 (PMC10930472; doi:10.3390/ani14050817)
Supplement: Supplementary file 1 [file animals-14-00817-s001.zip › Supplementary/Table S3.docx]

**Table S3**. Recategorisation needed for ordinal regression analyses.

| **Variable name** | **Original** | **Recategorised** |
| --- | --- | --- |
| Mucous membrane colour | Pink, Pale, Congested, Icteric, Cyanotic, Toxic line | Physiological, Pathological |
| Capillary Refill Time (CRT) | Normal, Slightly increased, Highly increased | Physiological, Pathological |
| Digital pulse | Not palpable, On one leg, On two legs, Palpable on all four legs | Not palpable, Palpable |
| Outcome | Discharged, Discharged not fully recovered, Euthanasia, Dead | Discharged, Euthanasia, Dead |
